# ‘Inchallah boza: between the forest, the bush and the town’: migrants’ language practices in Morocco

**DOI:** 10.1080/14797585.2025.2523744

**Published:** 2025-07-03

**Authors:** Mariangela Palladino, Anastasia Valassopoulos, Rajaa Essaghyry

**Affiliations:** a School of Humanities, Faculty of Humanities and Social Sciences, Keele University, Keele, UK; b The School of Arts, Languages, and Cultures, The University of Manchester, Manchester, UK; c RACINES aisbl, Belgium, Morocco

**Keywords:** Migration, language, mobility, translanguaging, communication, solidarity

## Abstract

Our study focuses on language practices among sub-Saharan migrants in Morocco by analysing the emergence, transformation and use of the words *tranquilo*, *boza* and *polo*. These terms signify place, movement and belonging and are important to explore migrants’ socio-political struggles. Our article foregrounds an analysis of these terms and language practices and offers nuanced understandings of experiences and lives in displacement. We argue that such language practices – besides performing social functions and meaning making – disrupt homogenous perceptions of migratory experiences and enable migrants to be active agents in the processes of cultural production, transmission, and transformation of language. This article intervenes in current debates on migrants’ linguistic practices and situates itself at the intersection of several fields of enquiry including migration studies, cultural studies, multilingual and language studies, and postcolonial studies.

## Introduction

Extant scholarship in migration studies has challenged categories, concepts and language (such as migrant, refugee, asylum seeker, camp, voluntary versus forced migration) often utilised by actors and institutions such as: the states, NGOs, etc. As Bakewell points out, ‘reliance of academic researchers on policy categories tends to obscure and render invisible some population groups, causal relationships, and questions that are methodologically difficult to capture’ (2011, p. 433). The last decade has seen an increase in scholarship which provides critical accounts on the need to examine how such categories are constructed. In the words of Crawley and Skleparis, this is a ‘body of academic literature that has demonstrated a disjuncture between conceptual and policy categories and the lived experiences of those on the move’ (Crawley & Skleparis, 2018, p. 59). Indeed, this is a protracted debate in migration studies on how such categories impact certain processes and how they are lived and embodied (Bakewell, 2011; Collyer & de Haas, 2012; Crawley & Skleparis, 2018; Gauci et al., 2015; Gupte & Mehta, 2007; Koser & Martin, 2011; Mainwaring & Brigden, 2016; Malkki, 1995; Richmond, 1993; Scherschel, 2011; Zetter, 2015). Social research offers numerous and vivid ethnographic accounts which pay close attention to the ways migrants call themselves, and how they make sense of their own experiences of displacement (Bachelet, 2018; Bachelet, 2025; Bredeloup, 2013; Vigh, 2009).

Crawley and Skleparis (2018) remind us that the categories dominating political, policy and media debates are inadequate and fail to encompass the complexities at the heart of migratory journeys. Thus they ‘perpetuate and reinforce a simplistic dichotomy which is used to distinguish, divide and discriminate between those on the move’ (p. 52). In the words of Bachelet (2019), ‘a focus on migrants’ emic terms enables us to engage more closely with the complex aspirations of migrants […] to be in a moving world where forces shaping one’s life are beyond one’s full control or understanding’ (p. 42). Indeed, research on the emergence and use of emic terms, an ‘insider’ language, among migrants and critical engagement with these, enables more nuanced understandings of their experiences. From ‘a lexicon of epic warfare (battles, attacks etc.)’ (p. 46), to warriors [‘guerriers qui frappent’] (Tyszler, 2018, p. 85) recent social studies shed light on language practices among those on the move. Such language practices include both migrants’ own self-representations (e.g. ‘adventurer’ Bachelet, 2025) as well as the ways they see and experience their movement and the places and spaces they inhabit.

There has been an increase in language and intercultural studies which focus on linguistic practices from speakers on the move; the literature which explores language in the context of migration focuses on multilingualism, translanguaging and belonging (Antonsich, 2010; Nagy, 2011; Gilmartin & Migge, 2016; Lamanna, 2021). Karatsareas terms it the ‘multilingual turn’ (2021). Experiences of migration provide a generative context for transforming language, or what Nagy defines as ‘contact-induced language change’ (Nagy, 2011, p. 1); other major areas of enquiry include fluid multilingual practices. When people move across the world, they draw on diverse repertoires of resources. This does not refer to simply drawing together ‘two separate languages nor to a synthesis of different language practices, or to a hybrid mixture. Rather, translanguaging refers to new language practices that make visible the complexity of language exchanges among people with different histories’ (Blackledge & Creese, 2018, p. 842).

This paper intervenes in current debates on migrants’ linguistic practices, it situates itself at the intersection of several fields of enquiry including migration studies, cultural studies, multilingual and language studies, and postcolonial studies. Based on qualitative data, our study focuses on migrants’ language practices by analysing the emergence, transformation and use of words which signify place, movement and belonging and are important for analysis of socio-political struggles over displacement – *tranquilo*, *boza* and *polo* – among sub-Saharan migrants in Morocco. This work proposes a shift in emphasis from previous research in social studies (on the significance of migrants’ lexicon to understand mobility issues) and in intercultural and language studies (centred on the linguistic dynamics taking place among speakers on the move). Its originality and contribution are twofold: its extensive analysis of the terms *tranquilo*, *boza* and *polo* as experienced and interpreted by migrants, and its focus on the cultural significance of migrants’ language practices. We reflect on the cultural processes implied in the use of these terms and how these become appropriated beyond the marginal, migrant community of speakers as they evolve across contexts. We foreground that such language practices – besides performing social functions and meaning making – disrupt homogenous perceptions of the journey and enable migrants to be active agents in the processes of cultural production, transmission, and transformation of language.

The article is structured as follows: we begin by outlining the context of our research with sub-Saharan migrants in Morocco. We offer a brief overview of our project MADAR – from which this study originates – before focusing on the project’s participants, our methods and approach to the study. The subsequent section offers an analysis of the data with a focus on the word *tranquilo* [used to describe places such as forests, houses and squats where migrants wait before crossing into Europe]; while engaging with existing literature, we examine our participants’ use, understandings and reflections on the word. We then move on to the word *boza* [meaning success or victory] which appears in our title too – as a key concept which captures migrants’ imaginaries of success; our analysis also explores *boza*’s linguistic influence beyond migrants’ contexts. *Polo* [a meeting spot, as well as location of destination or departure, related to and sometimes used interchangeably with *tranquilo*] features in the successive section which examines participants’ engagements with this term and its implications for place, movement and belonging. We bring our paper to a conclusion with reflections on the data and engagement with literature; finally, we offer a summary of our study’s contribution and its significance.

## Context

This paper is based on a study conducted at the heart of the project MADAR (Maghreb Action on Displacement and Rights 2020–2025) funded by the Arts and Humanities Research Council, UK Research and Innovation.^1^ The paper is grounded on our work within MADAR, and it specifically focuses on Morocco, one of the few countries across the Maghreb which has developed a migration policy (2013), known as the Stratégie Nationale de l’Immigration et de l’Asile au Maroc (SNIA – National Strategy for Immigration and Asylum in Morocco). This strategy included commitments to mobilise resources to promote protection and integration. While steps in the right direction have been taken, activists and civil society in Morocco (e.g. Racines, 2024) and internationally, still denounce racism, violence, denied access to health and education, and infringements of rights (including deportation) at the expense of sub-Saharan migrants, irrespective of their circumstances.

In this context, European border politics aimed at curbing migration (Schindel, 2022) play a key role in affecting social movements in southern Mediterranean countries (like Morocco). The politics and practices of protecting and policing EU’s external borders (Cobarrubias, 2020; Cuttita, 2022) such as the European Neighbourhood Policy (ENP) shifts the border control and border management on non-member states. Thus, countries often with inadequate human rights records, poor infrastructures, and scant migration legal and policy frameworks, control borders on behalf of the EU. Morocco’s geographical position and its proximity to Europe have rendered the country a hub for migrants from sub-Saharan Africa and for Moroccan themselves seeking to reach Europe irregularly (Berriane et al., 2015). The UNHCR reports (2025) the presence of 26,500 recognised refugees and asylum seekers in Morocco. The launch of the SNIA promised new instruments that would facilitate the integration of migrants in Morocco. However, it has failed to deliver effective changes. Moreover, Morocco’s partnerships with the EU trap migrants 'on the southern side of the Mediterranean for increasingly long periods of time in abject living conditions' (Bachelet & Palladino, 2023, p. 316). As such, the country’s migratory politics entail repression, co-option and control (Kamal, 2020; Kynsilehto, 2023). What scholars call transit migration (Bachelet, 2019) heightens migrants’ vulnerability as they live in dire conditions and exist in ‘a sort of legal limbo’ (Stock, 2019, p. 65) in which they see their rights violated.

Migrants make repeated attempts to seek entry into Europe from Morocco through non-linear migratory stages (Alioua, 2011; Cuttita, 2022) and repression which restrict their mobility; often they end up caught in a cycle of push-backs and forced removals at the hand of authorities. In these circumstances, sub-Saharan migrants in Morocco dwell in marginal and peripheral spaces (in urban areas, as well as in informal forest camps near the Spanish enclaves) as they organise (Gazzotti & Hagan, 2021) their attempts to cross the border. Our study focused on sub-Saharan migrants’ lived experiences in forest camps – in the north near the borders with Europe; across the centre where main urban areas are; and in the south, where many are forcibly displaced by authorities. These are makeshift encampments near the border zones at Ceuta and Melilla – the Spanish enclaves bordering Morocco to which migrants seek access; the camps are hiding places, while informal, these are very self-organised dwellings often structured around different nationalities (Escoffier, 2006; Jene 2010). They are set up by migrants themselves as they wait for the opportune moment to attempt border crossing. Significant border controls and repression by both Spanish and Moroccan authorities (Gazzotti, 2021, Natter, 2022) mean long waiting times in between attempts, thus migrants live in such informal camps during protracted periods and are exposed to extreme violence (Bachelet, 2025). The camps are also sites in which migrants are vulnerable (Stock, 2019), these are challenging spaces to dwell in and to access by researchers as well as civil society organisations.

## Methodology

The study sought to both document and shed light on migrants’ life in these forest camps, and it aimed to do so from migrants’ perspectives, through the production of a documentary film. The project was granted ethical clearance by the University of Keele (lead institution of the project) and all ethical considerations were thoroughly addressed. The methodology involved a participatory approach for two reasons: firstly, to place migrants at the centre of the process, and secondly, because accessing an informal migration camp (especially with cameras) was not feasible and not safe for researchers. Thus, in order to capture footage from within, we worked closely with those who actually dwelled in the camps. We approached NGOs working specifically on migration and forced displacement in Morocco, and sought access to people who lived in the camps and were willing to participate in the project. Initially, we interviewed 15 people, six women and nine men, aged between 20 and 50 years old, and from different countries including Senegal, the Ivory Coast, Cameroon, Ghana, Sudan, Nigeria, and Mali. The interviews took place across three different regions, in areas with a migrant population, namely in the centre (Casablanca-Settat), in the south, (Laâyoune-Sakia El Hamra), and in the north (Tangier-Tétouan-Al Hoceima). When approaching participants, we made sure that the project’s aims and methodology were clearly outlined. Our work took place amidst an ongoing debate on the ethical dimensions of art-based methods: mindful of what Mcllwaine and Ryburn have called ‘extractive knowledge’ (McIlwaine & Ryburn, 2024, p. 3324) practices, we sought however to deliberately engage with ‘a context in which discourse on migration is dominated by short-term political decision making and punitive policies (Jeffery et al., 2019, p. 13).

Three participants opted to be involved in the second stage of the data collection. We worked closely with them and co-developed the concept for the documentary. We learned about the living conditions within the camps, their internal organisational structures, the risks and dangers; such insights provided a knowledge base to draw up collectively an effective strategy (and relevant risk mitigation plan) to realise the documentary. In order to safely obtain footage from within the camps, we provided mobile phones to the three participants. Such an approach reduced risks for researchers, and also for participants themselves not to become hyper-visible, and be associated with outsiders. Camps are effectively hiding spaces, thus outsiders or intruders could compromise the safety of the camp and that of its dwellers. Before shooting, we trained participants on basic filming techniques to ensure optimum quality, in the style of Guerilla Cinema.^2^ Project’s participants living in the camp documented the daily life inside, they captured conversations among migrants, internal organisational dynamics, ordinary discussions, stories of hopes and resilience and the difficulties they faced within the camps and in general. Project’s participants were involved in the process from gestation to the very final editing, selecting footage, drafting interview scripts, and ultimately inscribed their own personal stories into the documentary.

The three participants were from Ghana, Guinea and Senegal, and their common goal was to transit through Morocco in order to reach Europe. Confronted with the difficulties involved in completing this mission, Morocco became a place of settlement. Participant 1 came to Morocco overland, from Accra; he has been on the road for 12 years. When he arrived in Morocco, he spent more than a year in an informal camp in Nador, at the border, attempting to reach Europe and pursue his dream of being a professional footballer. Faced with impenetrable barriers, he abandoned his dream in search of alternatives in Morocco. Participant 2 from Senegal, in his 30s, also arrived in Morocco on foot. After six months of living in informal camps, he made repeated unsuccessful attempts to cross into Europe. He said that he has given up, although he hopes that the roads to Europe will someday become more accessible. Meanwhile, he is working with an NGO that provides sub-Saharan migrants with urgent health assistance. As for participant 3, in his 30s, he first came to Morocco in 2009 by plane from Guinée Conakry, as part of a professional assignment, assisting junior soccer teams in different countries. He decided then to stay on in Morocco, where he forged relationships with a community of sub-Saharan migrants living in a disadvantaged district of Rabat (e.g. Attakadoum). He now works for an association that provides direct support to migrants and advocates for their rights. All participants could access the informal camps either through their contacts, and past experiences, or their current roles within the sub-Saharan migrant community. Participant 1 was interviewed in English whereas participants 2 and 3 were interviewed in French. Under MADAR’s aegis, the documentary titled *Boza! –* co-produced with the 3 participants – was completed in 2023 and has since been screened in different locations across Morocco, Europe, the US and has received several awards.

This paper focuses on a specific aspect of this project, namely, sub-Saharan migrants’ language practices. During initial meetings with NGOs and migrant communities, our exchanges with participants, as well as the film-making process pointed to a widespread usage of alternative terminology among migrants. The interviews – conducted face-to-face and online – provided extensive material and insights into a wide range^3^ of language practices including codes and terms utilised by migrants. During the interviews, participants were asked about these terms, their understanding of them, and when and where they had heard those words. Specifically, through interviews, we wanted to explore the ways in which interviewees engaged with these terms, the significance they attached to them, their appropriation of the words and how/if these words became important to them. Interviewees were also asked in what context they would use these terms and with whom. For example, it was important to have a sense of whether specific words were used when speaking with people from particular nationalities/communities and whether such terms might be known or used by the general public in Morocco. We were also keen to have a sense of whether all migrants understood these terms irrespective of country of origin and linguistic abilities. Such in-depth interviews delved into participants’ relations and usage of the terms, their experiences connected to such terms and their meaning, and how the terms might have changed or transformed over time. The transitory nature of migrant communities meant that much of this linguistic landscape was in flux and transmutable; we thus sought to better understand whether these words were used multimodally and whether their use had moved into more permanent artefacts (songs; films; proverbs and sayings). This paper focuses on three of the terms: *tranquilo*, *boza* and *polo*.

The interviews conducted function as an archive of a continuing and dynamic set of cultural formations evolving at speed. In order to best capture and appreciate the delicate appropriation of linguistic variance and to thoughtfully evaluate the work that it does, we here focus on the ways in which several particular words and phrases have morphed in order to reflect the changing migratory context. Our primary social context is that of mobility and how the transitional space that migrants may find themselves in, contingent as it is on specific social and material dynamics, brings about particular needs around communication. Being understood and ‘making sense’ is sometimes as narrow as the need that requires articulation. What a group of migrants seeking passage may have in common at any one time and who they might encounter and for what purposes, will dictate the particular temporal linguistic requirements: in other words, what needs to be communicated will be caught up in the creative maelstrom born out of precariously placed communities. Our interest broadly aligns with the position put forward by Gilmartin and Migge (2016) who argue that: ‘what bilingual and multilingual populations do with their linguistic resources [is influenced and] characterised by processes of mobility’ (p. 1). Specifically, we are interested in ‘how speakers draw on different languages in order to create social meaning’ (ibid).

## From the first to the last Tranquilo: home-making on the move

In thier work on the identity construction in contexts of migration, Gimartin & Migge shed light on the interconnected factors in operation that could influence and shape language use. Mobility brings about ‘in-between spaces’ that ‘affect mobile people’s language practices’ (Gilmartin & Migge, 2016, p. 5). These practices are of course based on a number of factors such as ‘personal, social and linguistic’ (p. 5) all of which may come to bear on how language is used across social practices: in other words what is said and how it is said. These ‘linkages between language and social practice’ (p. 5) reveal the extent to which language use is predicated on these social factors. When asked about certain words it was interesting to see how all participants coalesced around scenarios where terms appeared. These scenarios helped us to form a picture of migrants’ journeys and the situations and experiences that shaped them. Karatsareas’ work into linguistic variance in migration encounters also credits the multilingual turn with encouraging us to visualise languages not ‘as stable linguistic systems that are attached to particular geographical locations […] but as dynamic sets of semiotic resources that are owned by the people who use them in interactionally and contextually situated practices in order to make meaning, perform social functions, and negotiate identity positionings’ (Karatsareas, 2021, p. 2).

To the extent that we could set aside assumptions about how the migration journey might develop and the discourses that would influence this interpretation, we chose to compose our understanding of that journey based on the descriptions of the migrants and what they chose to highlight. Take *tranquilo* for example, a word that came up frequently during our discussions. From the ‘Spanish, meaning “rest” or “calm” [a] *tranquilo* is usually a small camp with three or four tents in the forest’ (Heck, 2011, p. 81). Participant 1 described it as a:

(a hideout) is where migrants are kept or hidden, those who wanted to cross the sea to go to Europe, it’s found in the bush before the forest, and after the town […] When I heard it, I was not sure, I met some migrants/passengers who had been in *tranquilo*, and were returning back home. They told me [a] lot of things about the journey, their stories, experiences, what was good and bad as well as about the *tranquilo*. So, this is how I had a clue about the circumstances during the journey and I was cool with it.

*Tranquilo* then, did not refer to a specific place, it did not stand in for the name of a town or area. In some cases the ‘*tranquilos* are mobile, which means that they can be taken down within minutes and set up in another area. In this way the migrants can escape the continuous persecution by the authorities’ (Heck, 2011, p. 81). For Lynes and El Montassir *tranquilos* are ‘sites of survival’ and function as ‘parallel societies’ (2020, p. 185). The *tranquilos* take ‘shape in relation to a changing social context and its attendant dangers’ (Lynes & El Montassir, 2020, p. 185). In our specific context they stood in for any place where one might wait, gather information, cultivate patience. Participant 2, originally interviewed in French (citations translated) reflected that:

The word *tranquilo*, as you know, is a word that migrants use a lot and when migrants say *tranquilo*, it’s their departure home, which we call *tranquilo*, it’s the house where we gather migrants to be able to make their journey; […] but *tranquilo* it came from Spain. So the moment the Spaniards rescue migrants on the sea, so they say *tranquilo tranquilo tranquilo tranquilo*, keep calm. Migrants have taken this word. They have defined this word in their hostel, now all the migrants living in ‘foyers’, sometimes twenty, call the ‘foyer’^4^ the tranquilo.

*Tranquilo* performs several functions here and works across contexts to both describe a place and a feeling. For Jean-Louis Edogué Ntang a *tranquilo* is a place deep inside the forest which offers more safety, where migrants feel secure. To get there, they would sometimes follow a very narrow pathway lined with deep pits, the bottom of which they could barely see in the dark (2023, p. 11).

‘Keep calm’ say the rescue workers in Spanish, but *tranquilo* is also a safe house where migrants await their departure. The instruction to ‘calm down’ in Spanish, in the social practice of waiting to leave however, comes to stand in for the place where one waits. The word then is transformed or is used to generate meaning out of the challenging conditions of waiting, fraught with uncertainty.

Although subject to some criticism for being a catch-all phrase (Wei 2018) Wei defends the term ‘translanguaging’ as a good theoretical model for a ‘theory of language practice’ as it captures ‘how social beings, with their diverse motives and their diverse intentions, make and transform the world’ (Wei 2018, p. 10). This making and transforming captures many of the contexts that we seek to engage with here. ‘Existing terms such as code-mixing and code-switching’ argues Wei, ‘seem unable to fully capture the creative and critical dimensions’ of expression borne out of new contexts (2018, p. 13). *Tranquilo* then is a shorthand across linguistic contexts and indicates access to a particular experience and also to a material place. It is also a rite of passage though, something that has to be understood and learnt but that also then opens the door to particular resources. Participant 2 reminisces on how he came to be introduced to the word and in so doing adapts his experience to incorporate this new terminology:

I remember when I was in Tangier, […] there is a guy, an Ivorian who said to me: ‘But is it that you know, do you know the *tranquilo* of Samba or the *tranquilo* of Demba?’ not to mention the name, I said it, said it, *tranquilo* de Samba, no I don’t know, I don’t know. He said oh okay, no problem. So, you have to come to the shop, once at the shop, you ask for Moussa’s *tranquilo* that we will show you, I said okay, there is no problem. Afterwards I stayed at home, I said to myself that ‘what is *tranquilo*? *tranquilo*s’, I said okay. So, it was the first time in Tangier that I heard this word *tranquilo* speak with the migrants there.

Though the word appears to be in frequent use, so much so that it has acquired geographical specificity (the *tranquilo* of Samba; the *tranquilo* of Demba), participant 2 had never heard the word before. The man from the Ivory Coast however assumes that he must know the word and that in some way, the word and its associated meanings, is relevant to him. The communication dynamics at play here are interesting – the word is not ‘native’ to either of these men. The man from the Ivory Coast however is further ahead in the journey and has already been inducted into the idiom of that particular point in the journey. Participant 2’s narrative performs his own participation into this new experience and is now in the ‘know’; he knows where to find the *tranquilo*s and who to ask if he cannot. The advantage here is that someone else has experienced the *tranquilo* before and can vouch for it, normalise it. In some ways the uncertainty of this already very precarious journey is made somewhat bearable through the sharing of this one word. ‘I was cool with it’ participant 1 reminds us.

Participant 2 elaborated on the extensive use of *tranquilo* as the place where migrants live, whether in a house or in the forest. If the house is for people who travel, it has become a *tranquilo*. The house then is transformed by its use *into* a *tranquilo*. Any house used by migrants who are waiting to travel is therefore a *tranquilo* and is understood as such by those who use it. As Heck observes, ‘some of these *tranquilo*s are mobile, which means that they can be taken down within minutes and set up in another area’ (2011, p. 81). This then gives the place a very different set of meanings that are shared by the communities accessing the *tranquilo* as a resource. This is expanded upon by participant 1 who noted that:

in *tranquilo* there are rules, regulations, and structures that everyone has to obey and follow them. The place is like a country, there are leaders like: the head, president, vice president, military, and even there’s a police station. If you’re wrong or you refuse to do what you’re told, you could be charged or punished in different ways for example: getting beaten, or doing certain jobs. If you’re dead you comfort yourself, it’s like a country, they rule.

This clearly refers to a much larger set up where the *tranquilo* operates as a hub with its own internal structures. This type of waiting space is institutionalised and is not the same as the bush, the forest, the foyer or the house. Yet, the same word is used for these spaces suggesting that although in some cases the understanding of *tranquilo* is contingent on its associative properties (large; crowded; lawless) another meaning overrides these specificities: the place where you wait before departure. This particular understanding seems to operate across any language and cultural barriers. As participant 1 notes, ‘everyone in [sic] the journey and on the road understands the word *tranquilo*.’ The word in this context transforms the relation of migrants to each other and also significantly impacts how they navigate the spaces nominated to be *tranquilo*s. Here the waiting occurs collectively and the objective is similar. Regardless of where the migrants have travelled from, they are travelling towards a similar end point. In order to arrive there, they must master *tranquilo*. ‘I started to use the word “*tranquilo*” in Mali in order to make friends, to integrate’ says participant 1.

Participant 2 also points out how *tranquilo* separates those who seek to migrate from those who assist in this enterprise:

I heard it [*tranquilo*] in 2009 for the first time. All the houses that were in Rabat or Casa where the migrants, the person who has to live, are there for the trip; we say there *tranquilo*. Because the place where the person who facilitates lives is not a *tranquilo* but where people travel, one or two or three mobilize, it has become a *tranquilo*.

To become a ‘*tranquilo*’ then, a space becomes designated as one through its use. Migrants seeking assistance and knowledge adapt their frames of references to, as participant 1 notes, fit in: if you can’t beat them join them.’ Participant 2 was also sanguine about the learning curve necessary in order to navigate new circumstances. In response to the researcher’s question on how they went about discovering new terms, it is clear that he ‘had to know what the codes are because that’s it. You have to first see the terrain and know how to use the same words.’ Understanding the terrain and working out the codes become part of reading this new context. Coming to terms with the significance of *tranquilo* also became apparent in conversations with participant 3. This includes learning how to find one’s way around the cities in Morocco in order to find the right people and places that could show you to the *tranquilo*:

You are told to go to the shop of, and you will ask there for the *tranquilo*, so these are Moroccan shops where they themselves know they know the *tranquilo*s, if you ask for the *tranquilo*, they go tell you that it’s Moussa’s *tranquilo*, that’s Demba’s *tranquilo*, it was in Tangiers.

We also learnt about variations in the type of *tranquilo*s available to migrants. These terms also worked to further clarify the stages of the journey and gave a temporal overview. Participant 2 was clear on how this functioned,

now there is another word also called ‘last *tranquilo*’, we use this word ‘last *tranquilo*’ […] the last *tranquilo* where people are mobilized because there are three types: the first *tranquilo* is where the others live; the second *tranquilo* is where people are sent before departure; now the last last *tranquilo* is by the sea, they are now waiting for the boats. We say: ‘where is he?, we are at the last *tranquilo*’.

Our study foregrounds much more nuanced understandings of *tranquilo* showing the different ways in which it is being mobilised across linguistic contexts, as space, as an affect, and as a stage – dramatising every aspect throughout the migratory experiences.

### ‘Boza, it’s like victory’: purpose, success and ontological status

Another significant term that has driven and shaped this study is *boza* (sometimes also spelt *bosa*) in the words of one of our participants ‘means that the migrants take the zodiacs and arrive in Spain, it’s the victory’ (1). The origins of the term are unclear: as Bajalia reminds us, ‘while some in the community point to *boza’*s origins in the Lingala word for ‘to have’ or ‘to be with’, others describe it as having emerged through a mixing of languages and terms, including *barzakh*, in Morocco’ (2023, p. 22). Bajalia also emphasises the important links of *al-barzak* to Islamic paradigms of pilgrimage (2023, p. 22). For Tyszler ‘the term is thought to come from Wolof or Bambara according to different explanations’ (Tyszler, 2019, p. 16). Bachelet states that among his informants ‘one of the words to denote crossing into Spain was “boza”. […] Although the etymology of the term is unclear, [my] Cameroonian informants explained that it meant “entering” in Cameroonian slang but had become a synonym for “victory” and getting into Europe’ (2019, p. 19). Participant 2 offered us a specific and elaborate definition: ‘the word *boza* refers to when migrants manage to gain access, to achieve their objective. The objective is to enter any Spanish territory, whether by road, by sea or through the barriers’. This definition makes reference to the context – migrants in Morocco – to place – stepping on Spanish soil – and to a specific goal: reaching the objective. Bajalia notes that *boza* is an ‘indeterminate process without a clear beginning or end. […] we may consider boza as both a proposition (I am making *boza*) and a thing (a successful trip across the border, for instance)’ (2023, p. 23). Participant 1 also elaborates further on the strict relationship between *boza* and the objective: ‘if the objective is reached, they say *boza*, it’s like victory, it’s like the objective is reached, etc. It’s like a team that has scored goals, it’s like saying Morocco has *boza* a goal; it means they’ve scored a goal. This word is attributed to victory, to reaching the goal’.

This quote powerfully deploys a metaphor from football – which represents a pervasive imaginary among migrants – to articulate *boza* in all its nuances as reaching the objective as success and victory implied in stepping on European soil and fulfilling their migratory dream. Participant 3 shares with us *boza*’s significance as a state of being and on the level of rights or prospect of access to rights; recalling when he enquired about the meaning of the word the first time, he said: ‘when I asked others, they said *boza* it’s those who entered, those who have freedom now […]. So *boza* it’s the convoy that succeeded!’. There are three elements from this quotation which are worth highlighting. The first one relates to the notion of entering: ‘those who enter’ suggests that those setting foot beyond the frontier into Europe have somewhat gained access to an exclusive (and exclusionary) place. The word freedom unmistakably marks out those who enter from those who do not: entering means becoming free, it represents in their imaginary a changing station, it is a rather ontological dimension, afforded by *boza*. This resonates with Bajalia’s ethnographic work which foregrounds ‘making boza’ as ‘an indeterminate process. It is not clear when it begins or ends. Boza is both an event (crossing the fence or the sea) and a proposition (’I am making boza’) that describes an active, ongoing, present progressive tense of being’ (Bajalia, 2023, p. 30). Such present progressive tense of being is what we describe as an ontological dimension. The reference to ‘convoy’ in the participant’s quotation brings in a register drawn from military contexts: the success of the convoy highlights not only that crossing borders irregularly with the aim to reach Europe is experienced by migrants as a military operation (there are months or years behind an attempt to cross by sea or scale the fence in Ceuta and Melilla – the inland Spanish enclaves) but also because convoys suggest collectivity and organisation which are key to migrants’ journeys and experiences.

The project’s participants were all very well aware of the first time they used the word *boza* and offered insightful perspectives about origins, use, and emergence of the term. Participants 1 stated that ‘*boza* has dominated everywhere because it’s known by everyone, especially the francophone migrants, and I first heard it here in Morocco.’ The specificity of *boza* to the Moroccan context and migrant population was highlighted by all participants. Participant 2 noted ‘I first heard the word *boza* in 2009 when I arrived for the first time in Morocco’. Participant 3 shared with us that he ‘heard *boza* in Tangier. Once when I was there, I heard that they say that such a person did *boza*, or such a pirogue did *boza*, or such and such a convoy did *boza*’. In this statement too, the collective nature of *boza* as an operation – with reference to pirogue and convoy – is unmistakably foregrounded alongside its military connotations.

Participant 2 situates the surfacing of the term more specifically in time and in relation to a key moment in migration history in Morocco which brought that land border to the international limelight: ‘It’s a word that came out in 2005 during the mass entry of migrants into Ceuta and Melilla’. This refers to the 2005 incident when the border fence (between Spain and Morocco) in Ceuta was scaled by hundreds of migrants who attempted to reach Spanish soil; the Spanish and Moroccan police used rubber bullets and live ammunition to control the border, and this resulted in a number of migrants dead and wounded.

The same participant when asked about the origin of the term was also generous in his articulation of the generative context and not just the timeframe: ‘everyone uses this word without knowing its origin or the real meaning of the word. […] One of my friends […] he spent 22 years in Morocco […] he was part of this movement of people who came up with this word because it’s a word that comes out directly between people and everyone uses it’. For this participant *boza* is generated, among other words, by a group of people – rather a movement – which suggests purposeful organisation and collective action. Karastsareas reminds us that ‘language varieties have multiple owners and the complex links between them are under constant negotiation and readjustment’ (Karatsareas, 2021, p. 2). The ‘comes out directly’ suggests an organic generative vernacular process followed by a similarly organic and seamless adoption of the term ‘everyone uses it’. Understanding of the term seems to be followed by actively using it: ‘I started using it because when you’re part of the environment, you’re going to pick up words, […] these words influence everyone, when you’re in the community and they’re speaking, you memorise too’.

This participant lucidly shared with us how he began deploying the term, there is a consciousness about the active use of the term and being part of a broader ecology. Moreover, the participant also stresses how such words actually influence and shape the vernacular landscape and propagate among an increasingly wider pool of speakers. Thibault notes that individuals ‘adapt their bodies and brain to the languageing activities that surround them’ (Thibault, 2017, p. 76). This results in what is aptly termed a ‘linguistics of participation’ approach (Wei 2018, p. 15) or what Naomi Nagy has called ‘contact-induced language change’ (Nagy). This can help us to frame the emergence, use and transformation of words and phrases in the context of sub-Saharan transitory migrant communities in Morocco. Karastsareas points to the use of language in these contexts as a tool with which to ‘make meaning, perform social functions, and negotiate identity positionings’ (Karatsareas, 2021, p. 2).

There is an actual process of transmission taking place here with reference to *boza* from a long-standing member of the migrant community to migrants newly established in Morocco: ‘because the elders already had this word, it’s a code’. The reference to the code here is not incidental, *boza* ‘comes directly to people as a code’, it is understood as a term that allows secrecy, enables belonging to a specific group with similar circumstances and with the same goal, *boza*. This echoes what Antonsich called ‘the politics of belonging’ in migrant communities who find themselves in transitory social and linguistic spaces (2010). Specifically, it is the means through which ‘they create place-belongingness’ (Antonsich 2010) and how this intersects with the creation and use of insider vernaculars (Lamanna 2021) to result in the appropriation and subsequent utilisation of new words for transitional contexts. Participant 3 reminds us of the context in which *boza* is used: ‘Yes, because we were all migrants and we all had the same goal; it was to leave here and go to Europe; so all we talked about these words, *tranquilo*, *boza*, […] all the time.’ This quotation illustrates even more overtly the term *boza* in relation to its speakers’ shared circumstances and its purpose: a code that is limited (but not only) to a group of people who seek to maintain a level of secrecy about their communications.

*Boza* has not remained a code among migrants only, it has spread to others living, working or dealing with migrants, including activists from the EU; referring to Moroccans, participant 3 said: ‘they start to know the word as well; because they say that maybe the sub-Saharans who are there, they’re there to *boza*.’ Moroccans’ use of the word is not limited to identifying or characterising migrants and their purpose and goal while in Morocco – crossing the border to reach Europe – it has moved from use to actual re-appropriation. Participant 2 explains that: ‘those who live with migrants, those who are around migrants. Even internally displaced Moroccans, when you meet them, will say “Inchallah *boza*”, they use the word’. The phrase ‘Inchallah *boza*’ encapsulates at once a word which is transient and specific to the migrant population in Morocco (*boza*), and an expression of hope and wish (Inchallah, God willing) in Arabic used in everyday life by Moroccans. The phrase brings together two distinct realities – the migrant and host communities with fraught racial relations – and reconciles them into a shared language which captures hopes, dreams and wishes. Lamanna refers to generative contexts such as in displacement and explores ‘insider vernaculars’, which, along with ‘translanguaging’ indicates that situations where communities generally operate within two or more languages and in certain contexts they ‘freely select from the specific features of this language according to their communicative needs in specific contexts’ (Lamanna, 2021, p. 1).

*Boza* has outgrown the circle of speakers and somewhat lost its status as a code; not only it is used and adapted by speakers outside the migrant community, but it has also been transposed as a term and applied to other contexts. 'Not just in the context of migration. […] I told myself, it’s a word that reaches the objective, the goal […] I say that they *boza*. […] it means that you got married, you *boza*, you’re admitted, you *boza*. I now try to employ these words every time'. As participant 3 points out, *boza* is known widely now, there already exists an informal archive on social media about *boza*, known as the cry of victory; referring to personal footage of migrants who reach the European union, he commented: ‘the videos that you see, the migrants scream *boza*, they make signs, and today on social media, everyone is aware of these keywords.’ *Boza* has already left a digital footprint, it is – as the participant pointed out – a key word that at once expresses victory, a purpose, an ontological status (a migrant seeking access into Europe). This study contributes to an expansive understanding of *boza* which signifies all of these; it is about a state of being, about collectivity, it is part of a broader ecology which has moved from being a code to an adopted linguistic practice. For participant 3 it is simply ‘beautiful. […] Yes, it’s freedom, it’s liberté’, it represents so much in two syllables.

### ‘Polo 14’: placemaking for distance and destination

*Polo* is another key term that emerges from the interviews and footage. It is distinct from the other terms in its exclusive connection to space, which may be accounted for by the differentiated character of migrants’ experiences with movement or displacement. One of the meanings of *polo* is a gathering spot for migrants, mainly for begging. Bachelet notes that ‘*polo* designates a favourable situation or place in Cameroonian street slang; *polo* was also used by sub-Saharan migrants to designate a hideout’ (2019, p. 860). The reference to Cameroonian street slang is the only trace we identified about the derivation of the term, and during our interviews, none of the participants elaborated on the origins of the word – even when prompted. Participant 2 explains: ‘*polo* means, for example, the place where migrants and children beg, we say it’s a good *polo* […] If you join (the begging network), you may know the word *polo*’. It is used in urban contexts to indicate topographical markers such as traffic lights, shopping centres, bus stations: ‘at the traffic lights there is the *polo* of such and such, ah it’s our *polo*’. In the context of street begging, the *polo*s are not only places of mixed communities, but also of mixed ages and genders. ‘If migrants go to the Marjane centre to beg, for example, they’ll say it’s a good *polo*. There’s the Guinean women’s *polo*, there’s the so-and-so *polo*, it’s over there’. A *polo* is a specific spot, a corner; like *tranquilo*, it has a fluid meaning with connotation to space, place and their social function.

Most data, however, suggest that *polo* signifies knowledge, proficiency, and almost mastery of certain routes, either by newcomers or indeed by those repeatedly attempting to cross into Europe. Participants refer to the term *polo* either as a meeting point or as a departure location. Participant #2 explains that ‘in the forest there are *polo* where people make attempts’. Participant 3 claims that *polo* refers to ‘the places where migrants make their departures. You’re going to hear, *polo* 14, *polo* 7; because *polo* 14 means it’s in this place of departure, in this position it’s 14 kilometres from Spain. So, if you say *polo* 7, it’s 7 km from Spain on the sea, that’s Tangier’. This quotation refers to the distance between Morocco and Spain, specifically, the 14 km of sea which separate the two countries and indeed continents. This stretch of sea represents the shortest – yet not safest – route into Europe by sea which migrants seek to cross. Thus, *polo* 7 in the citation above, refers to a specific location in the journey to *boza*!, it is halfway between departure and arrival. *Polo* here appears to function as a set of coordinates (7; 14) legible by migrants on a particular point of the journey. Importantly, the *polo* was changeable depending on the circumstances that would enable the journey to take place. These act like staging points on a complex route where only certain communities are aware of the information at any given point. Accuracy is crucial and reflects the importance of the reliability of the information supplied in a migratory context. This is a determining factor in the lives of migrants for several reasons. Migrants might fail in their attempt to make the crossing if they do not know the exact *polo* and therefore risk the *boza* and may lose the money paid for the crossing. Other problems can also arise from not being in the correct *polo*. For example, ‘if an Ivorian finds himself in the *polo* of a Cameroonian and is found to be begging there, he might run into trouble’. Participant 2 notes that: ‘we say here it’s the *polo* of the Nigerians, for example the barrier, that’s the *polo* of the people of Gourougou […] You can’t get there without going through them, you can’t come like that’. Clearly, there are rules that govern and oversee this space that work separately from official or more formal structures. Nevertheless, these are broadly understood to be necessary, and compliance is expected. Partly this serves to provide some sense of rootedness, even if this is temporary. We noticed that the term *polo* is also used by migrants as a tool, as participant 3 declares: ‘yes, we use *polo* to locate ourselves, if it’s the forest, we use *polo* to locate the point of their departure.’

*Polo*s are never neutral places, and participants would always add an adjective qualifying the *polo*s (good, not good) according to what they can bring in terms of money, but also according to the presence of the authorities there. Although often they are closed and exclusive to particular communities for a long time, in some cases *polo*s are also places of social mixing where different communities gather in harmony. Participant 2 shared that: 'now, here it’s mixed, you can see everyone, Guineans, Senegalese, Cameroonians, they go to the same place, same traffic lights. For example, the traffic lights there is the *polo* of such and such, ah it’s our *polo'*. While referring to a period where there was no social mixing in a specific *polo*, participant 2 stated that: ‘before there was dictatorship, here it’s the Cameroonians, no one can come, now it’s changed’. This observation reflects the changing nature of migrants’ social spaces; this may be attributed to various factors, including religious and cultural affinities and sometimes pressure exerted by the authorities on some particular sites which in turn drives people away.

Knowledge of the word *polo*, like for the others above, signifies access to networks, information and ultimately experience gathered from repeatedly seeking entry into Europe. As claimed by participant 2, ‘if you’re not an elder, you can’t understand the word *polo*’. This citation not only alludes to experience as outlined above, it also refers to hierarchies within migrant communities and ultimately places value to particular forms of knowledge. It somewhat legitimises such transient and marginal epistemologies. We were reminded that: ‘the elders can say “The *polo* there was good, because there were no authorities there”, it didn’t work but the *polo* was good, “Hmm the *polo* wasn’t good because the bums there attacked us”’. Once again, the reference to elders signifies such recognition of knowledge, but also points to these figures which contribute to generating knowledge and also decision making. Building on existing literature of *polo* (e.g. Bachelet, 2019), this paper surfaces alternative readings of the term whereby it signifies knowledge and proficiencies of certain sites and where it functions differently from *tranquilo –* not only as a place, but also as a reference point to map out routes and movements.

## Conclusion

As social functions and identity positions shift and as the reasons for pursuing certain productive meanings in light of changing needs and desires transform, so too language becomes ‘deterritorialised’ as ‘everyday occurrences’ change radically. Events and experiences accrued through migratory experiences and expedited mobility require new means of expression and fresh forms of participation, including language acquisition. Wei (2018) argues that ‘language learning is a process of embodied participation and resemiotization’ (17), a position shared among other socio-linguists. This embodied participation points to an understanding of language as mutable, bending and flexing according to context. ‘Language then’ writes Wei, ‘is a multisensory and multimodal semiotic system interconnected with other *identifiable* but *inseparable* cognitive systems’ (Wei 2018, 20). It is this new ‘translanguaging space’ that Wei argues is responsible for ‘genereat[ing] new configurations of language’ (24). This frame of the ‘translanguaging space’ has been effective in allowing us to explore the ways in which migrants in Morocco engage in productive and rich communication strategies.

In addition to this, we also remain sensitive and alert to the linguistic practices of the participants and their interaction with the practices of researchers both of whom ‘develop certain strategies based on their linguistic biography and migratory experiences’ none of which are ‘independent of language hierarchies’ (Havlin, 2022, p. 2). Relationships between researchers and participants, and the communication that goes into building these, feature significantly in this article as we strive to better understand the frameworks within which translanguaging takes place and worked diligently not to ‘exacerbate concerns around ownership, anonymity, confidentiality and representation’ (McIlwaine & Ryburn, 2024, p. 3236)

The located, highly specific and often transitory practices that we have outlined, give shape to an evolving experience and provide insights into how this is articulated over time. We foreground the emergent languages practices of these communities and the labours by which they are maintained, and reflect on how ‘complex communicative tasks and demanding environments’ arise (Wei 2018, p. 25). Our work has contributed to reconceptualise how we might ‘understand migration as the outcome of socially produced knowledge’ (Stielike & Löhr, 2025, 2508). This article has also engaged an interdisciplinary approach in its exploration of terms agentively deployed by migrants in contexts of mobility – *boza*, *polo* and *tranquilo*. By recording these transient linguistic practices, and engaging with speakers (in this case also project’s participants), we foreground nuanced analysis of the terms and offer new insights into their use and significance across linguistic contexts. The participants offered up opportunities for dynamic engagement on how they utilised *boza, tranquilo* and *polo* to make sense of and give shape to experiences of vulnerability, mobility and community in displacement.

## Notes

1. MADAR sought to address the humanitarian protection of vulnerable, displaced people in the context of conflict in the Maghreb, specifically in Algeria, Morocco and Tunisia. Involving academic institutions across the UK and the Maghreb as well as non-governmental organisations as research partners, this cross-sector, interdisciplinary, international project addressed displacement in the Central Maghreb by focusing on diverse areas of enquiry (including but not limited to: experiences of displacement; migrant women’s health; migrant minors’ access to education; etc.).

2. Guerrilla Cinema is about making films on a limited budget by creatively exploiting public spaces, with minimal crews and basic equipment. It generally involves filming in public places without the necessary permits. Refer to the project for further details: Reel Borders https://reelborders.eu/Home

3. These include but are not limited to: *eke* (police); *kpakey* (arrest); *ekeray* (street beggar); *shayo* (drinking alcohol); *polo* (departure point; meeting point with traffickers); *tranquilo* (camp, house); *boza* (adventure, crossing, managing to cross the sea).

4. The word ‘foyer’ here refers to crowded housing that is largely self-managed by migrant communities.
